# *Sphingobacterium multivorum* cellulitis: case report and mini-review

**DOI:** 10.1016/j.nmni.2024.101502

**Published:** 2024-10-10

**Authors:** Matteo Coen, Aurélie Foulex, Ilias Bagetakos, Abdessalam Cherkaoui, Jacques Serratrice, Jacques Schrenzel, Anne Iten

**Affiliations:** a Service of Internal Medicine, Department of Medicine, Geneva University Hospitals, Geneva, Switzerland; b University of Geneva, Faculty of Medicine, Unit of Development and Research in Medical Education (UDREM), Geneva, Switzerland; c Division of Primary Care Medicine, Department of Primary Care Medicine, Geneva University Hospitals, Geneva, Switzerland; d Service of Radiology, Clinique Générale-Beaulieu, Geneva, Switzerland; e Service of Radiology, Department of Radiology, Geneva University Hospitals Geneva, Geneva, Switzerland; f Bacteriology Laboratory, Service of Laboratory Medicine, Department of Diagnostics, Geneva University Hospitals, Geneva, Switzerland; g Service of Infectious Diseases, Department of Medicine, Geneva University Hospitals, Geneva, Switzerland; h Infection Control Program, Geneva University Hospitals, Geneva, Switzerland

**Keywords:** Non-fermenting gram-negative bacteria, Gardening, Soil, Immunocompromised patients, Antimicrobial resistance

## Abstract

**Background:**

*Sphingobacterium multivorum* is a gram-negative, non-fermentative, oxidase-positive, catalase-positive bacillus. *S multivorum* has been identified in urinary tract infections, respiratory tract infections, spontaneous peritonitis, septic arthritis, meningitis, bacteraemia and septic shock. Ours is the second case of skin and soft tissue infection sustained by *S. multivorum* (a case of necrotizing fasciitis with septic shock has been previously reported). In this paper, we furnish a review of the literature on all the cases of *S multivorum* described in the medical literature (with the different antimicrobial susceptibility profiles for each case).

**Case presentation:**

We describe the case of a dermo-hypodermitis of the right arm, forearm, and postero-lateral abdominal wall sustained by *S multivorum*. The infection occurred in an 84-year-old woman with a medical history of type 2 diabetes, chronic kidney disease, and refractory psoriatic arthritis treated with tocilizumab.

**Discussion:**

*S multivorum* is a ubiquitous gram-negative bacillus, characterized by a variable antibiotic susceptibility profile that is difficult to anticipate.

**Conclusion:**

*S multivorum* is an opportunistic pathogen capable of causing rare but potentially severe infections in patients of all age groups, with a higher prevalence in immunocompromised individuals, as observed in our case.".

## 1 Background

*Sphingobacterium multivorum,* first described in 1981 [1], is a gram-negative, obligate aerobe, non-fermentative, oxidase-positive, catalase-positive bacillus. It belongs to the genus *Sphingobacterium*, which houses bacteria containing large amounts of sphingophospholipids in their membranes [2]. Previously classified as *Flavobacterium multivorum* because of its capacity to produce yellow pigments (from Latin *flavus*, yellow), *S. multivorum* is ubiquitous [3] and previously considered a contaminant of the public transport system [4]. Initially designated as non-pathogenic [5], several human infections have been reported since the 1980s (Table 1) [6]. Two additional cases were reported by Blahova et al. [7].

**Table 1:** ***Sphingobacterium multivorum* cases published in the literature**.

Reference	Sex/Age	Country	Comorbidities	Causes	Diagnosis	Samples	Treatment	Outcome
Dhawan [6] 1980	M/60 y	USA	Alcoholic liver disease		Spontaneous bacterial peritonitis	Peritoneal fluid	Ampicillin and gentamicin, then carbenicillin	Full recovery
Potvliege [15] 1984	M/43 y	Belgium	Hemodialysis	Puncture of the fistula? Dialysate?	Bacteraemia	Blood	Ampicillin and tobramycin	Full recovery
Freney [16] 1987	M/57 y	India	Immunoblastic-type non-Hodgkin's lymphoma	Hospital drinking water?	Bacteraemia	Blood	Perfloxacin then TMP/SMX	Full recovery
Reina [21] 1992	F/20 m	Spain	Cystic fibrosis		Acute exacerbation of chronic bronchopathy	Bronchial aspirates	Ceftazidim and amikacin	Full recovery
Aydogan [18] 1993	M/2 m	Turkey	Healthy	Sub-optimal hygiene condition (earthquake)? Cutaneous scratch?	Septic shock	Blood	Ampicillin and cefotaxim	Full recovery
Areekul [22] 1996	M/47 y	Thailand	Diabetes mellitus HIV infection	Not defined	Respiratory infection and bacteraemia	Sputum and blood	Ampicillin and gentamycin, then ceftriaxone and TMP/SMX	Death
Vella [23] 2001	M/74 y	Spain	Chronic obstructive pulmonary disease		Respiratory infection and bacteraemia	Bronchial aspirates	Ceftazidime, then cefuroxime	Full recovery
Lambiase [24] 2009	F/<22 y	Italy	Cystic fibrosis Pancreatic insufficiency	Not defined	Chronic pulmonary infection; co-infection	Sputum	No precision	No deterioration of lung function
Lambiase [24] 2009	F/<22 y	Italy	Cystic fibrosis Pancreatic insufficiency	Not defined	Chronic pulmonary infection; co-infection	Sputum	No precision	No deterioration of lung function
Lambiase [24] 2009	M/<22 y	Italy	Cystic fibrosis Pancreatic insufficiency	Not defined	Chronic pulmonary infection; co-infection	Sputum	No precision	No deterioration of lung function
Grimaldi [11] 2012	F/64 y	France	Rheumatoid arthritis treated with steroids Obesity Diabetes mellitus Coronary heart disease	Dog scratch	Necrotizing fasciitis and septic shock	Fascia and Subcutaneous tissues	Amoxicillin + clavulanate	Full recovery
Nielsen [20] a 2014	M/79 y	Denmark	Renal insufficiency: hemodialysis Prostate cancer	Transrectal ultrasound-guided prostate biopsy (environment via foam pads and biopsy needle)	Urinary tract infection and bacteraemia	Urine and blood	Piperacillin + tazobactam and ciprofloxacin	Full recovery
Nielsen [20] b 2014	M/59 y	Denmark		Transrectal ultrasound-guided prostate biopsy (environment via foam pads and biopsy needle)	Cystitis	Urine	No antibiotic treatment	Full recovery
Nielsen [20] b 2014	M/69 y	Denmark	Enlarged prostate	Transrectal ultrasound-guided prostate biopsy (environment via foam pads and biopsy needle)	Cystitis	Urine	Trimethoprim	Full recovery
Barahona [19] 2015	F/67 y	USA	Obesity, dyslipidaemia, hypertension Diabetes mellitus Chronic obstructive pulmonary disease Obstructive sleep apnoea Pulmonary hypertension	Water or soil of rehabilitation facility	Septic shock	Blood	Cefepime and vancomycin, then ciprofloxacin	Full recovery
Mendes [113] 2015	M/6 y	Brazil	Liver transplant due to biliary atresia		Septic arthritis (knee)	Joint fluid	Oxacillin and ceftriaxone, then ciprofloxacin	Full recovery
Abro [14] 2016	M/28 y	United Arab Emirates	Healthy	Skin injury acquired during a field exercise	Acute meningitis and bacteraemia	Blood	Ceftriaxone	Full recovery
Pardavila [10] 2019	F/75 y	Spain	Active seropositive rheumatoid arthritis Diabetes mellitus, hypertension Coronary heart disease, paroxysmal AF Hypothyroidism	Pressure ulcer	Infection of pressure ulcer	Exsudate from infected pressure ulcer	Ciprofloxacin	Full recovery
Konala 12] 2020	F/70 y	USA	Multiple myeloma in remission Hyperlipidaemia, hypertension Hypothyroidism Chronic obstructive pulmonary disease	Dog injury	Cellulitis	Blood	Levofloxacin	Full recovery
Highton [9∗] 2021	M/6 y	Argentina	Healthy	Burn injury	Skin and soft tissue infection	Tissue culture	Meropenem	Full recovery
Muzzafar [17∗] 2023	M/40 y	India	Diabetes mellitus	Grade IV infected sacral bedsore	Bacteraemia	Blood	TMP/SMX	No information
**Coen [this paper]** **2024**	F/84 y	Switzerland	Psoriatic arthritis treated with tocilizumab Diabetes mellitus Chronic kidney disease	Skin injury acquired during gardening	Cellulitis and bacteraemia	Blood	Amoxicillin + clavulanate, then TMP/SMX then meropenem	Death

Abbreviations.

M Male.

F Female.

Yyear

Mmonth

AFatrial fibrillation.

TMP/SMXTrimethoprim –sulfamethoxazole.

## 2 Case presentation

**Fig. 1:**
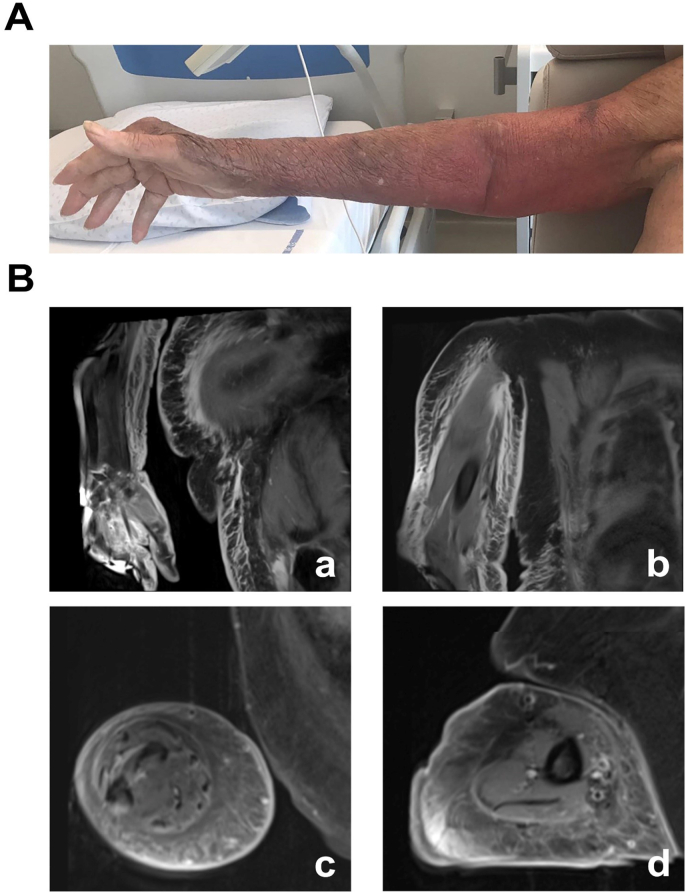
A. Erythematous lesion of the inner side of the upper arm. B. MRI images showing diffuse dermo-hypodermitis of the right arm and forearm associated to superficial fasciitis of the *biceps* and *triceps brachialis* as well as of *extensor* and *flexor carpi ulnaris*.

An 84-year-old woman was admitted for profound fatigue and orthostatic hypotension that occurred a few days after gardening bare-handed at home; she sustained no injuries during the gardening. She had a history of type 2 diabetes, chronic kidney disease, and refractory psoriatic arthritis (with hand lesions), treated with tocilizumab. Two days after admission, her condition worsened with fever and the appearance of a swollen erythematous lesion of the right upper arm (Fig. 1A). Laboratory findings showed leukocytosis (14.5 G/l), thrombocytopenia (109 G/l), and a slight CRP increase (29.90 mg/l). According to the quick Sequential Organ Failure Assessment score [8], the patient did not meet the sepsis criteria. Ultrasonography showed synovitis of the *extensor carpi ulnaris* tendon with infiltration of the subcutaneous tissues. Two sets of blood cultures were obtained before antibiotics administration. Empirical antibiotics (IV amoxicillin/clavulanate, 1.2 gr/12h) were then started.

Bacterial growth was detected in the two aerobic bottles after 7 h of incubation in the BD BACTEC™ FX system. MALDI-TOF MS (Bruker Daltonics) identified *S. multivorum* with a score value > 2. Minimum inhibitory concentrations (MIC) were determined using the E-test method according to the manufacturer's instructions (bioMérieux). *S. multivorum* isolate was susceptible to trimethoprim/sulfamethoxazole (TMP/SMX), meropenem, and levofloxacin (MIC = 0.064, 1.5 and 0.750 mg/L, respectively). Detailed antibiotic susceptibility testing results (Table 2) revealed a complex pattern.

**Table 2:** **Reported antibiotic susceptibility profiles of *Sphingobacterium multivorum***.

References	Dhawan [6]	Potvliege [15]	Freney [16]	Reina [21]	Aydogan [18]	Areekul [22]	Vella [23]	Lambiase [24]	Grimaldi [11]	Nielsen [20] a	Nielsen [20] b	Barahona [19]	Mendes [13]	Abro [14]	Pardavila [10]	Konala [12]	Highton [9]	Muzzafar [17]	Coen [pub]
	1980	1984	1987	1992	1993	1996	2001	2009	2012	2014	2014	2015	2015	2016	2019	2020	2021	2023	2024
Ampicillin	R	R	>32		I	R				R	R			R		R			
Ampicillin/sulbactam					S										R	R			
Amoxicillin – clavulanate					S				S					I					
Piperacillin	S		128	S	I			R				I		R	R				R
Piperacillin-tazobactam								R		R	R		R		R	R		S	R
Ticarcillin-clavulanate				S	S		I		S						R				
Cefuroxime		R		S	S		S			R	R								
Cefotaxime			16	S	S	S		R				S	I	S	R				
Ceftriaxone			8	S	I	S						S	S	S		R			
Ceftazidime			16	S	R	S	R	R				R		I	R	R		R	R
Cefepime								R				S	S	S					S
Imipenem				S	S		S	R				S	R	R	R	R		R	R
Meropenem								R				I	R		R		S		S
Amikacin	R	R	>32	S	S	R		R				S	R	R	R			R	R
Gentamicin	R	S	>16	R	S	R		R		R	R	S	R	R	R	R			S
Tobramycin	R	R	>16	R	R		I						R		R	R			R
Tetracycline	S	S	2		S	S	S					S		S					
Chloramphenicol	S	S	8	S		S		R											
Ciprofloxacin				S	S			S		S	S	S	S	S	S	S	S		R
Levofloxacin								S					S		S	S	S	S	S
Trimethoprim-sulfamethoxazole	S	S	5	R	R	S		S	S	S	S	R	S	S	S	S			S

MICs in mg/L.

Despite therapy, cellulitis extended to the arm over 48 h. An MRI concluded a diffuse dermo-hypodermitis of the right arm, forearm, and postero-lateral abdominal wall, as well as fasciitis of the biceps and triceps brachialis and of the extensors and flexors of the carpus without osteomyelitis (Fig. 1B).

Amoxicillin/clavulanate treatment was stopped and meropenem treatment initiated (1.5 gr/12h) with rapid regression of cellulitis. On day 3, the treatment was simplified to oral trimethoprim/sulfamethoxazole (TMP/SMX) (800/160 mg/24h) due to the patient's challenging venous access and the high sensitivity of *S. multivorum* to TMP/SMX. The renal function deteriorated rapidly. The decline in kidney function was believed to be due to a prerenal cause, supported by a favorable Fractional Excretion of Sodium (FeNa) and Fractional Excretion of Urea (FeUrea), and the absence of signs indicating intrinsic or post-renal causes. Moreover, kidney function demonstrated slight improvement with volume repletion. However, since acute kidney injury can be linked to the use of TMP/SMX, we chose to take a cautious approach and switched back to meropenem for the rest of the treatment 14 days of total antibiotic treatment). To note, blood cultures were obtained one week after initiating effective antibiotic treatment, and they came back negative.

Due to the patient's worsening condition, which included severe chronic pain and several comorbidities, she made the decision to stop receiving treatment intended to extend her life. She was moved to the palliative care unit, where she passed away shortly afterwards of causes unrelated to *S. multivorum* infection.

## 4 Discussion

*S. multivorum* has been identified in urinary tract infections [9], respiratory tract infections [[10], [11], [12], [13]], spontaneous peritonitis [6], superinfected wound^9^∗^10^∗^,^ cellulitis ^11,12^∗, septic arthritis [13], necrotizing fasciitis [11], meningitis [14], bacteraemia [[15], [16], [17]] and septic shock without any apparent source of infection [18,19]. Ours is the third skin and soft tissue infection sustained by *S. multivorum*. Grimaldi et al. have reported a case of necrotizing fasciitis with septic shock [15].

All age groups can develop *S. multivorum* (range two months-84 years old; Table 1).^6, 9, 11-24,^ *S. multivorum* most often affects individuals with risk factors like immunosuppression [[11], [12], [13],^16^,^22^] and comorbidities [6,11,15,[19], [20], [21], [22], [23], [24]]. *S. multivorum* is an environmental bacterium. Cases are reported from North America [6,12,19], South America [9,13], Southeast Asia [16,17,22], Europe [10,11,15,18,20,21,23,24] and the eastern Mediterranean area [14]. *S. multivorum* infections are acquired following cutaneous lesions [[9], [10], [11],^18^], dog-related skin lesions [12], accidents [14], precarious hygiene conditions [18]. The quality of water [16,19] and soil maintenance [19] have been questioned in hospitals and rehabilitation facilities. *S. multivorum* can potentially cause healthcare–associated infections [9,10,17,20]. Nielsen et coll [20]. were alerted by inoculation of *S. multivorum* in the prostate by prostate biopsies; the use of non-sterile material was incriminated, and revision of the urological procedure solved the problem. In a hemodialysis center, water or dialysis water baths or contamination from the dialysate through an undetectable leak in the dialyzer membrane were suspected [15]. The source often remains unidentified [22,24] or not mentioned [6,13,21,23]. *Sphingobacterium* spp. can survive in moist environments and contaminate laboratory culture media [19].

The patient in our case report presented multiple predisposing factors (psoriasis, diabetes, chronic kidney disease, immunosuppression). She was likely infected while gardening through a breach in the skin barrier.

*Sphingobacterium* spp. are intrinsically resistant to many antibiotics; moreover, *S. multivorum* can produce an extended-spectrum–β-lactamase and a metallo-β-lactamase conferring resistance to third-generation cephalosporins and carbapenems respectively [3,7,22]. The antibiogram susceptibility profile varies among isolates without a typical susceptibility pattern (Table 2). TMP/SMX, quinolones, and tetracyclines are the most common susceptible therapies. Given the rarity of these infections, potential severity, and diverse antimicrobial susceptibility profiles, it is essential to rapidly identify *S. multivorum* and introduce appropriate treatment. Antibiotic susceptibility testing (Table 2) revealed that the isolate was resistant to piperacillin-tazobactam, ceftazidime, and imipenem but susceptible to cefepime and meropenem. Among aminoglycosides, resistance was observed for amikacin and tobramycin, but gentamicin remained susceptible. Discrepant results were obtained for quinolones (ciprofloxacin resistance levofloxacin susceptibility). Therefore, empiric therapy should contain a combination of drugs (e.g., a carbapenem and fluoroquinolone) to minimize treatment failure risks before obtaining the isolated organism's antimicrobial susceptibility profile.

## 5 Conclusions

*S. multivorum* is a ubiquitous bacterium which can cause serious infections. Until now, the adequate management of these infections has been delayed by the time required to obtain bacteriological identification and antibiotic resistance profile. Available microbiological tools can shorten these steps and permit adequate targeted therapy. Wearing protective equipment adapted to the activities (gloves, shoes, and clothing), regular maintenance of the environment of the accommodation premises, and respect for hygiene recommendations during medical procedures should make it possible to avoid such infections.

## Availability of data and materials

Data sharing does not apply to this article as no datasets were generated or analyzed during the current study.

## CRediT authorship contribution statement

**Matteo Coen:** Writing – review & editing, Writing – original draft, Investigation, Formal analysis, Data curation, Conceptualization. **Aurélie Foulex:** Writing – review & editing, Data curation, Conceptualization. **Ilias Bagetakos:** Writing – review & editing, Data curation, Conceptualization. **Abdessalam Cherkaoui:** Writing – review & editing, Data curation, Conceptualization. **Jacques Serratrice:** Writing – review & editing, Writing – original draft, Data curation, Conceptualization. **Jacques Schrenzel:** Writing – review & editing, Writing – original draft, Data curation, Conceptualization. **Anne Iten:** Writing – review & editing, Writing – original draft, Formal analysis, Data curation, Conceptualization.

## 3 Search strategy

To achieve a comprehensive understanding of the diverse clinical manifestations of *S. multivorum*, an extensive research effort was undertaken. A review was conducted with no restrictions on publication dates, covering literature from the inception of the PubMed database through September 2024. This approach aimed to gather relevant articles detailing various clinical presentations and infections associated with this microorganism. The search strategy was meticulously developed to ensure a thorough review of the literature. We employed a structured approach using a range of keywords and Medical Subject Headings (MeSH) terms, such as “*Sphingobacterium multivorum*”, “infection”, “cutaneous infection”, and “sepsis”. This strategy was designed to encompass a wide spectrum of clinical manifestations and pathological presentations related to *S. multivorum*. The review sought to provide an in-depth analysis of *S. multivorum* infections, including both common and rare presentations. By integrating MeSH terms and specific keywords, we aimed to deliver a detailed and nuanced understanding of the pathogen's role in infectious diseases. We included seminal studies from the 1980s, as well as recent case reports, to reflect the evolution of knowledge and the latest developments in the field. In addition to examining clinical manifestations, the research focused on identifying trends in treatment approaches, including patterns of antibiotic resistance and effective therapeutic options. By synthesizing information from a broad range of sources, the review aimed to enhance our understanding of *S. multivorum*'s impact on various patient populations and provide valuable insights for clinicians managing infections caused by this pathogen.

## Ethics approval and consent to participate

An ethical waiver was obtained from the Ethics Committee of Geneva.

Consent for publication was obtained from the patient (before her death). Moreover, the patient's next of kin has given written permission to publish her relative's clinical details and images.

## Funding source(s)

Not applicable.

## Declaration of competing interest

The Authors declare no competing interests.

## Abbreviations

**MRI**: Magnetic Resonance Imaging
**MALDI-TOF/MS**: Matrix-Assisted Laser Desorption Ionization-Time of Flight Mass Spectrometry
**TMP/SMX**: trimethoprim/sulfamethoxazole
**MIC**: minimum inhibitory concentration
